# The relationship of pre-procedural Dmax based sizing to lesion level outcomes in Absorb BVS and Xience EES treated patients in the AIDA trial

**DOI:** 10.1007/s10554-019-01576-y

**Published:** 2019-03-25

**Authors:** Ruben Y. G. Tijssen, Laura S. M. Kerkmeijer, Yuki Katagiri, Robin P. Kraak, Kuniaki Takahashi, Norihiro Kogame, Ply Chichareon, Rodrigo Modolo, Taku Asano, Martina Nassif, Deborah N. Kalkman, Yohei Sotomi, Carlos Collet, Sjoerd H. Hofma, Rene J. van der Schaaf, E. Karin Arkenbout, Auke P. J. D. Weevers, Marcel A. M. Beijk, Jan J. Piek, Jan G. P. Tijssen, Jose P. Henriques, Robbert J. de Winter, Yoshinobu Onuma, Patrick W. Serruys, Joanna J. Wykrzykowska

**Affiliations:** 1 Heart Center, Department of Clinical and Experimental Cardiology, Amsterdam Cardiovascular Sciences, Amsterdam UMC, University of Amsterdam, Amsterdam, The Netherlands; 2 The Department of Cardiology, Onze Lieve Vrouwe Gasthuis, Amsterdam, The Netherlands; 3 The Department of Cardiology, Medical Center Leeuwarden, Leeuwarden, The Netherlands; 4 The Department of Cardiology, Tergooi Hospital, Blaricum, The Netherlands; 5 The Department of Cardiology, Albert Schweitzer Hospital, Dordrecht, The Netherlands; 6 ThoraxCenter, Erasmus Medical Center, Rotterdam, The Netherlands; 7 NHLI, Imperial College London, London, UK

**Keywords:** Bioresorbable scaffolds, Quantitative coronary angiography, Scaffold thrombosis, Target lesion revascularization, Device sizing

## Abstract

Due to expansion limits of the Absorb bioresorbable scaffold a meticulous implantation with correct sizing is required. We sought to investigate the clinical outcomes based on the sizing of the device related to the maximal lumen diameter measured by quantitative coronary angiography in Absorb BVS and Xience EES treated lesions in the AIDA trial. Sizing of Absorb bioresorbable vascular scaffold (BVS) and Xience everolimus eluting stent (EES) was graded according to the definitions of device non-oversize and device oversize on pre-procedural angiography. Lesion-oriented outcomes (LOCE) (device thrombosis, TLR and TVMI) that occurred during 2 years follow-up were related to device non-oversized or oversized status. In the Absorb BVS group, LOCE occurred in 48 (7.4%) lesions in the oversized group and in 32 (8.2%) lesions in the non-oversized group (HR 0.91; 95% CI 0.58–1.42; p = 0.681), whereas TLR occurred in 34 (5.3%) lesions and in 23 lesions (5.9%), respectively (HR 0.89; 95% CI 0.52–1.51; p = 0.666). Definite scaffold thrombosis occurred in 11 (1.7%) device oversized treated lesions against 16 (4.1%) device non-oversized treated lesions (HR 0.41; 95% CI 0.19–0.89; p = 0.020). There were no differences in event rates between oversized and non-oversized groups in lesions treated with Xience EES. There was no significant difference in LOCE between oversized and non-oversized treated Absorb BVS and Xience EES treated lesions. Non-oversized Absorb BVS implantation was associated with a higher risk of scaffold thrombosis at complete 2 years follow-up. The majority of very late scaffold thrombosis occurred in properly sized devices.

**Electronic supplementary material:**

The online version of this article (10.1007/s10554-019-01576-y) contains supplementary material, which is available to authorized users.

## Introduction

The early results of Absorb everolimus-eluting bioresorbable vascular scaffold (Absorb BVS) implantation in simple coronary lesions were promising [1–3]. Initial enthusiasm was hampered when large randomized trials with long-term follow-up reported higher rates of target lesion failure (TLF), and scaffold thrombosis in the Absorb BVS compared to Xience everolimus eluting stent (EES) [4–7].

The limitations of the device, as well as implantation techniques or strategies, have been proposed as possible causes of adverse events [8]. Proper sizing of the scaffold may be of great importance acutely after scaffold implantation. The limited ability to over-expand makes the Absorb BVS prone to scaffold disruption when an undersized device is implanted and aggressively post-dilated. Furthermore, implanting an oversized device has been shown to cause more edge dissections and peri-procedural myocardial infarctions [9–11].

Ishibashi et al. demonstrated that implantation of an oversized Absorb BVS was associated with a higher risk of major adverse cardiac events (MACE) at 1 year [12]. Whereas, Katagiri et al. demonstrated that implantation of an undersized Absorb BVS was associated with a higher risk of MACE between 1 and 3 years [13]. This association was investigated in trials, which included only simple lesions and where quantitative assessment of the target vessel diameter by online quantitative coronary angiography (QCA) was required for enrollment in the trial [1–3, 14].

The Amsterdam Investigator-Initiated Absorb Strategy All-comers (AIDA) trial was an all-comers randomized controlled trial that analyzed Absorb BVS versus Xience EES in routine percutaneous coronary intervention (PCI). Although it demonstrated non-inferiority in the composite endpoint of target vessel failure (TVF) of Absorb BVS versus Xience EES at 2 years, Absorb BVS was associated with a higher risk of definite/probable device thrombosis [15]. In the current report we investigate whether proper sizing is a key factor leading to adverse events after device implantation and therefore we evaluated the relationship of maximum lumen diameter (Dmax) based device sizing on QCA, to lesion oriented outcomes in both the Absorb BVS and Xience EES arms of the AIDA trial.

## Methods

### Study design AIDA trial

The AIDA compared Absorb BVS (Abbott Vascular, Santa Clara, CA, USA) with Xience EES (Abbott Vascular, Santa Clara, CA, USA) in routine PCI. The study design [16], the preliminary safety report [6], and the 2-year results [15] have been published previously.

Briefly, between August 2013 and December 2015, 1845 consecutive patients with coronary artery disease undergoing PCI with one or more target lesions suitable for drug-eluting stent implantation were included in AIDA. Key exclusion criteria were target lesions longer than 70 mm, a visually estimated reference vessel diameter of < 2.5 mm of > 4.0 mm, treatment of a true bifurcation lesion with a priori planned two device strategy, and treatment of in-stent restenosis. Patients were randomized after successful pre-dilatation of the first lesion. Device sizing was based on visual assessment by the operator. Online QCA, or periprocedural testing of cardiac biomarkers, were not mandatory. Device implantation strategy was planned according to the instructions for use of the implanted device.

### Design of the current analysis

#### QCA analysis

The current analysis includes all study lesions treated with at least one study device of which baseline angiogram suited for offline QCA analysis was available. QCA assessment of device sizing relied on the angiographic diameter function curve of the pre-treatment vessel segment consisting of three non-ambiguous luminal dimensions; namely, the minimal lumen diameter (MLD) and the Dmax in the proximal segment (proximal Dmax) and in the distal segment of interest (distal Dmax) with respect to the MLD.

Pre- and post-procedural measurements were performed in either (1) multiple matched views or (2) a single matched view. If no matched views were available, measurements were done within the view with visually the highest stenosis grade. Proximal and distal Dmax were measured in (a) pre-procedural projection(s). In case of a total occlusion, the MLD was considered to be 0 mm; the proximal Dmax was measured in a pre-procedural projection; and the distal Dmax was considered similar to the proximal Dmax. Offline QCA analyses were initially performed on the post-procedural angiograms within the Absorb BVS arm, and were performed by seven experienced readers [6]. We later added 5 experienced readers to our academic Corelab, in order to complete pre-procedural measures on the Absorb BVS arm, and complete the pre-procedural and post-procedural measures within the Xience EES arm. All QCA analyses were performed using Cardiovascular Angiography Analyses System [CAAS], version 5.11 (Pie Medical Imaging, Maastricht, the Netherlands). All QCA readers were blinded for clinical events, and were supervised by one cardiologist [YO], who is an expert in this field.

#### Definitions

The study population in both arms was stratified by the difference between both the proximal and distal angiographic Dmax and the nominal diameter of the implanted device. The selection of device size was considered “oversized” when the proximal and distal Dmax of the treated lesion were smaller than the nominal size of the device. Lesions with either a proximal or a distal Dmax or both Dmax larger than the nominal size of the device constituted the “non-oversized group”. When a patient received 2 or 3 overlapping devices in a long lesion, the nominal size of the proximally implanted device was compared with the proximal Dmax, whereas the nominal size of the distally implanted device was compared with the distal Dmax. Devices were considered properly sized when the ratio of the proximal or distal Dmax minus the device diameter fell within the range of -0.5 to + 0.5 mm.

#### Lesion oriented endpoints

The lesion-oriented composite outcome (LOCE) of this analysis included definite device thrombosis, target lesion revascularization (TLR) and target vessel myocardial infarction (TVMI). All myocardial infarctions were defined by the Third Universal Myocardial Infarction definitions and other events were defined according to the Academic Research Consortium definitions. All reported events were adjudicated by an independent Clinical Events Committee (Cardialysis BV., Rotterdam, the Netherlands).

### Statistical analysis

This report provides information on the lesion-oriented outcomes subdivided by their device non-oversize or oversize status. All analyses were conducted using the per-protocol population. Descriptive statistics were used to present the data. Groups were compared using Chi square test and student t-test. Kaplan–Meier method, log-rank test and Cox proportional hazards models were used for time-to-event analysis. Landmark analyses were performed at 31 days and 365 days after index procedure. A 2-sided p value < 0.05 was considered significant. The prognostic value for LOCE of device oversize was assessed in a univariate Cox proportional-hazard analysis. In a multivariate Cox regression analysis we identified other statistically significant predictors of LOCE. Next we performed a multivariate regression analysis for device oversize with adjustment for other statistically significant predictors of LOCE.

The entry criterium for the multivariable analysis was set at p < 0.1. Device oversize was forced into the multivariate model. All statistical tests were performed using SPSS software, version 23.0 (IBM SPSS Statistics, IBM Chicago, IL, USA).

## Results

**Fig. 1:**
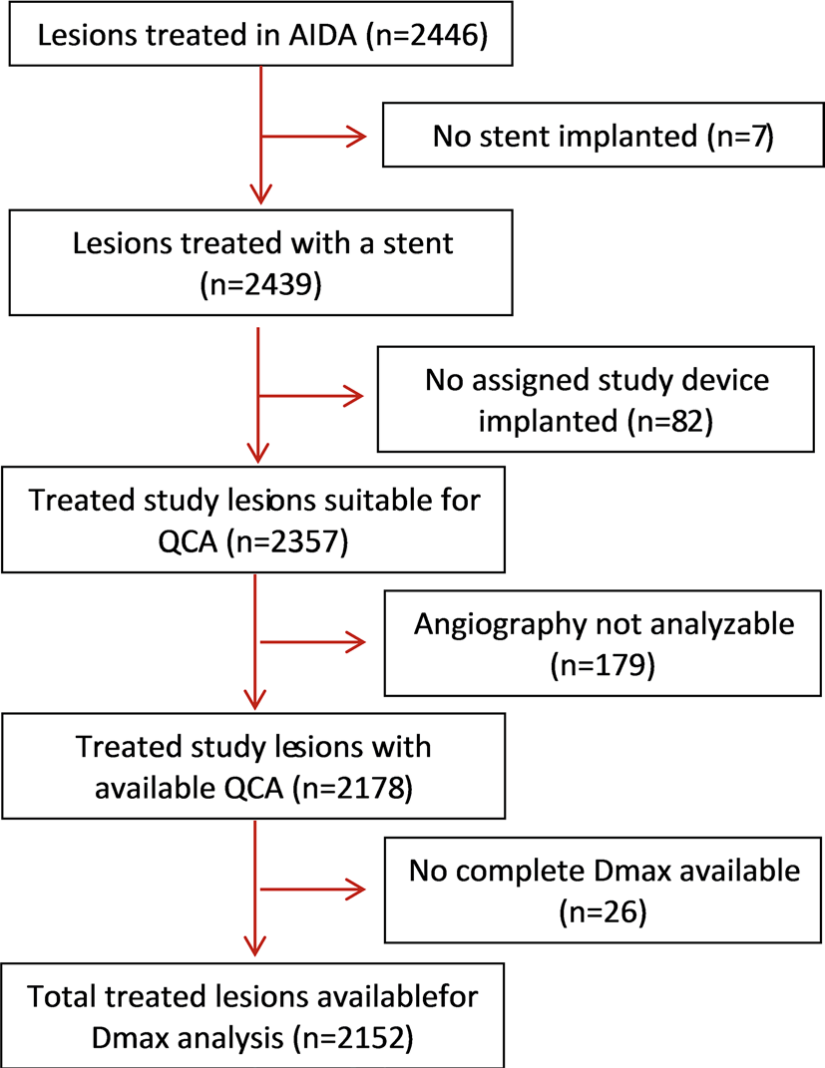
The study flowchart. *AIDA* Amsterdam Investigator-Initiated Absorb Strategy All-comers, *QCA* quantitative coronary angiography, *Dmax* maximum diameter

In the AIDA trial, 924 patients were randomized to treatment with Absorb BVS and 921 patients to treatment with Xience EES. A total of 2446 lesions were treated; 1237 lesions within the Absorb BVS arm and 1209 within the Xience EES arm. We excluded 89 lesions that did not receive any study device. Another 179 lesion were not analyzable, and were therefore excluded. Pre-procedural Dmax assessment was available in 2152 lesions (87.9%). The full study flowchart is shown in Fig. 1.

Baseline and angiographic characteristics of the oversized and non-oversized groups for both study devices are displayed in Tables 1 and 2. In 653 (62.0%) of Absorb BVS treated lesions, and 648 (59.0%) of Xience EES treated lesions an oversized device was implanted. Oversized devices were more frequently implanted in the left coronary artery, and in lesions with a TIMI flow of 0/1. In both study devices pre-procedural reference vessel diameter (RVD), both proximal and distal Dmax and minimal lumen diameter (MLD), were significantly smaller in the oversized groups compared with the non-oversized groups. Moreover, pre- and post-dilatation were less frequently performed in the Absorb BVS oversized group compared with the non-oversized groups.

**Table 1:**
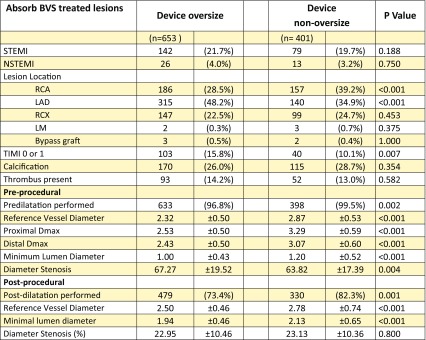
Shown are the clinical and angiographic characteristics of Absorb BVS treated lesions, stratified by oversized/non-oversized device

Plus-minus values are means ± SD

*BVS* bioresorbable vascular scaffold, *(N)STEMI* (non)ST-elevation myocardial infarction, *RCA* right coronary artery, *LAD* left anterior descending coronary artery, *RCX* ramus circumflex, *TIMI* thrombolysis in myocardial infarction, *Dmax* maximum diameter

**Table 2:** Shown are the clinical and angiographic characteristics of Xience EES treated lesions, stratified by oversized/non-oversized device

Xience EES treated lesions	Device oversize	Device non-oversize	P Value
	(n = 648)	(n = 450)	
STEMI	151 (23.3%)	82 (18.2%)	0.010
NSTEMI	26 (4.0%)	22 (4.9%)	0.502
Lesion location			
RCA	178 (27.5%)	143 (31.8%)	0.138
LAD	304 (46.9%)	172 (38.2%)	0.004
RCX	161 (24.8%)	130 (28.9%)	0.144
LM	2 (0.3%)	4 (0.9%)	0.234
Bypass graft	3 (0.5%)	1(0.2%)	0.648
TIMI 0 or 1	109 (16.8%)	36 (8.1%)	0.001
Calcification	170 (26.2%)	128	0.448
Thrombus present	102 (15.7%)	42 (71.6%)	0.002
Pre-procedural			
Predilatation performed	607 (93.7%)	406 (90.2%)	0.039
Reference vessel diameter	2.31 ± 0.57	2.79 ± 0.59	< 0.001
Proximal Dmax	2.58 ± 0.50	3.24 ± 0.65	< 0.001
Distal Dmax	2.50 ± 0.49	3.04 ± 0.65	< 0.001
Minimum lumen diameter	0.94 ± 0.37	1.09 ± 0.46	< 0.001
Diameter stenosis	68.77 ± 18.67	66.06 ± 16.85	< 0.001
Post-procedural			
Post-dilatation performed	295 (45.5%)	243(54.0%)	0.008
Reference vessel diameter	2.54 ± 0.50	2.76 ± 0.57	< 0.001
Minimal lumen diameter	1.88 ± 0.48	2.06 ± 0.51	< 0.001
Diameter stenosis (%)	26.23 ± 11.68	25.30 ± 11.11	0.189

Plus-minus values are means ± SD

*EES* everolimus eluting stent, *(N)STEMI* (non)ST-elevation myocardial infarction, *RCA* right coronary artery, *LAD* left anterior descending coronary artery, *RCX* ramus circumflex, *TIMI* thrombolysis in myocardial infarction, *Dmax* maximum diameter

### Lesion oriented outcomes

Lesion-oriented outcomes are shown in Table 3. LOCE occurred in 48 (7.4%) lesions in the Absorb BVS group oversized group and in 32 (8.2%) lesions in the non-oversized group (HR 0.91; 95%CI 0.58–1.42; p = 0.681).

**Table 3:**
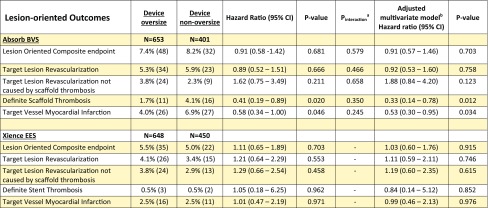
Shown are the lesion oriented outcomes at 2 years of Absorb BVS and Xience EES treated lesions

*BVS* bioresorbable vascular scaffold, *EES* everolimus eluting stent, *CI* confidence interval

^a^Interaction for device oversize and device modality

^b^Adjusted for age, male sex and Syntax score for Absorb BVS, and adjusted for hypertension, hypercholesterolemia, any diabetes mellitus, and moderate to severe calcification for Xience EES

**Fig. 2:**
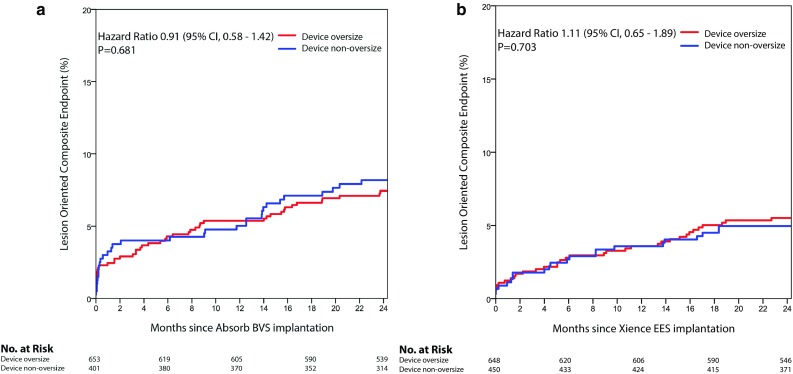
Shown are the event rates of the lesion oriented composite endpoint in oversized or non-oversized Absorb BVS (**a**) and Xience EES (**b**) treated lesions. *BVS* bioresorbable vascular scaffold, *EES* everolimus eluting stent, *CI* confidence interval, *no*. number

**Fig. 3:**
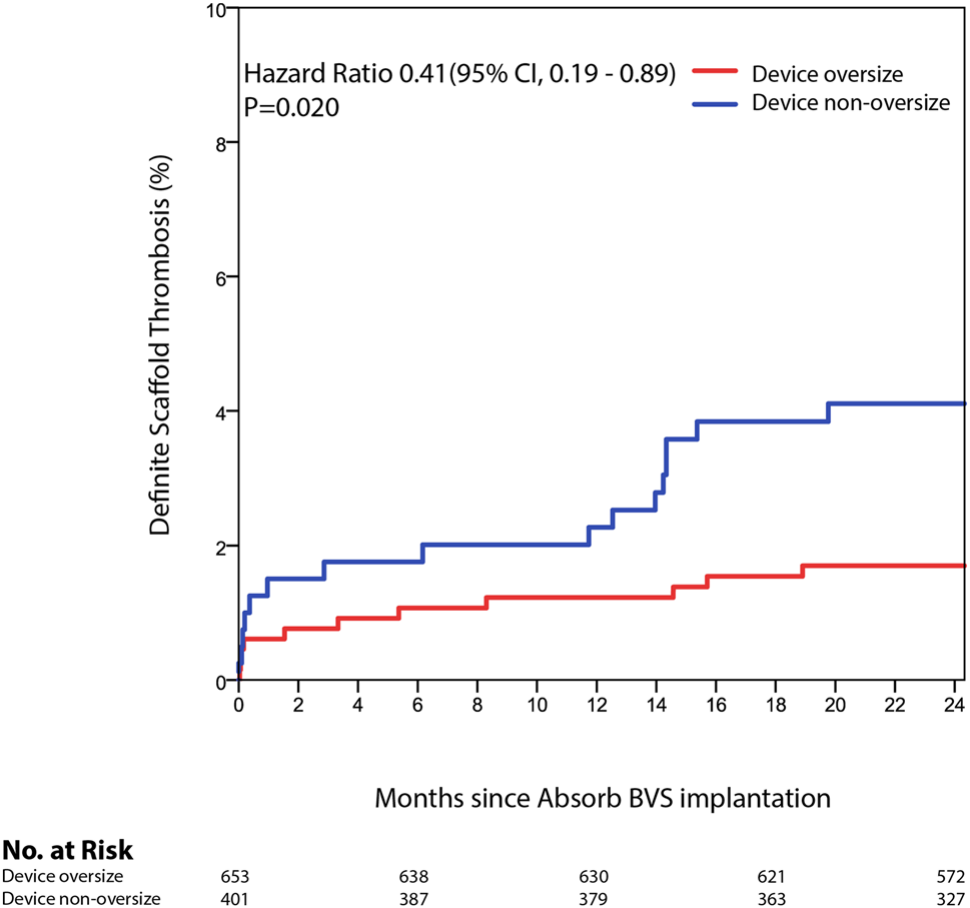
Shown are the event rates of definite scaffold thrombosis in oversized or non-oversized Absorb BVS treated lesions. *BVS* bioresorbable vascular scaffold, *CI* confidence interval, *no*. number

LOCE occurred in 35 (5.5%) in the Xience EES group oversized group and in 22 (5.0%) lesions in the non-oversized group (HR 1.11; 95% CI 0.65–1.89; p = 0.703). Kaplan–Meier curves for LOCE at 2 years after Absorb BVS or Xience EES implantation are shown in Figs. 2, 3, respectively.

**Fig. 4:**
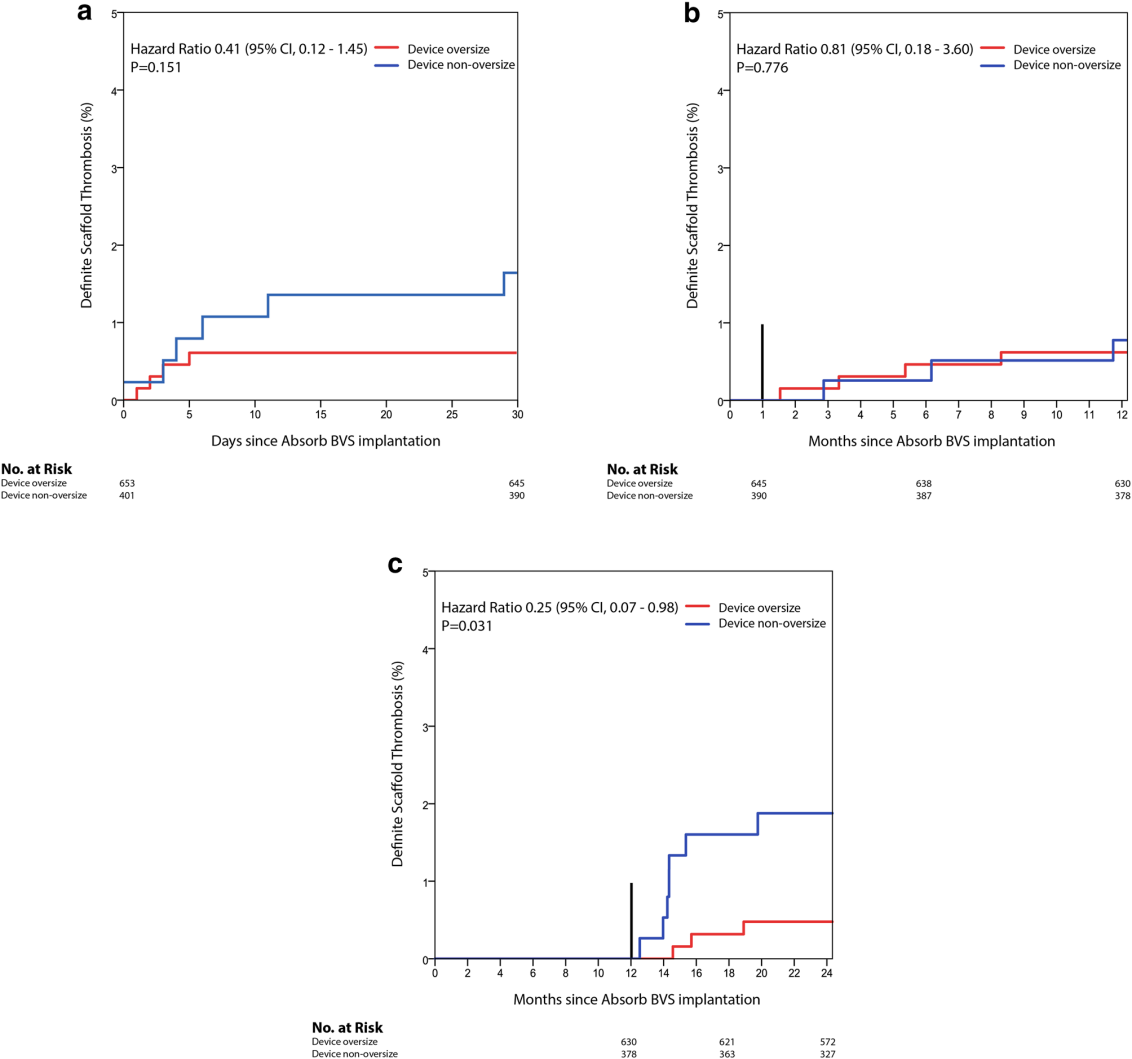
Landmark analysis of definite scaffold thrombosis at 31 days (**a**), 31 to 365 days (**b**) and 1–2 years (**c**). *BVS* bioresorbable vascular scaffold, *CI* confidence interval, *no*. number

**Fig. 5:**
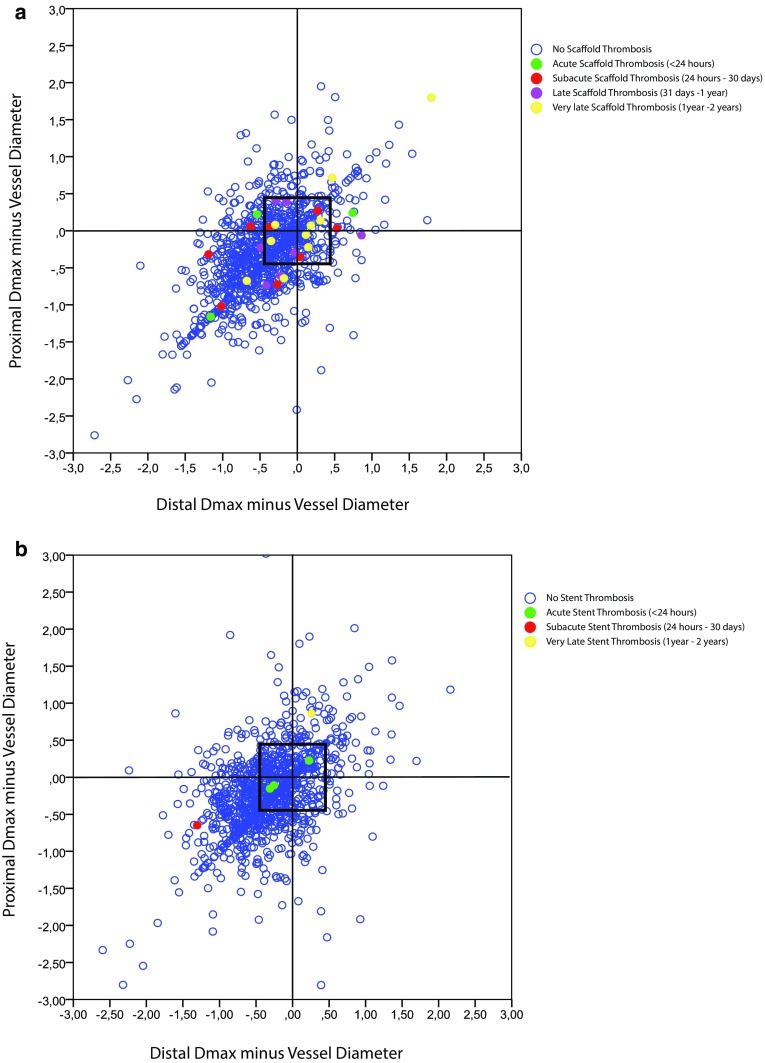
Shown is the distribution of proximal and distal Dmax minus the nominal device size in Absorb BVS (**a**) or Xience EES (**b**) treated lesions with or without definite device thrombosis. *BVS* bioresorbable vascular scaffold, *Dmax* maximum diameter, *EES* everolimus eluting stent

Within the Absorb BVS arm TLR occurred in 34 (5.3%) lesions in the device oversized group and in 23 lesions (5.9%) in the device non-oversized group (HR 0.89; 95% CI 0.52–1.51; p = 0.666). TLR not caused by scaffold thrombosis occurred at higher, but non-significantly different, rates in the device oversized group (HR 1.62; 95% CI 0.75–3.49; p = 0.211). Definite scaffold thrombosis occurred in 11 (1.7%) device oversized treated lesions against 16 (4.1%) device non-oversized treated lesions (HR 0.41; 95% CI 0.19–0.89; p = 0.020) (Fig. 3). Landmark analyses of definite scaffold thrombosis until 31-days, from 31 days to 1 year, and from 1 to 2 years after scaffold implantation are shown in Fig. 4a–c. Definite scaffold thrombosis from 1 to 2 years occurred less frequently in oversized Absorb BVS treated lesions (HR 0.25; 95% CI 0.07–0.98; p = 0.031). Figure 5a, b show explorative analysis of the distribution of proximal and distal Dmax minus the nominal device size in Absorb BVS treated lesions (A) and Xience EES treated lesions (B) with or without definite device thrombosis. The majority of very late scaffold thrombosis cases occurred in properly sized scaffolds that fell within the definitions of the Instructions for Use of the Absorb BVS and the Dmax assessment. Supplementary Figs. 1 and 2 show the ROC curves of the relative difference between the Dmax and the device diameter and LOCE (1) and definite device thrombosis (2) at 2 years after Absorb BVS or Xience EES implantation.

The predictors of LOCE at 2 years after Absorb BVS or Xience EES implantation are shown in supplementary table 1 and 2, respectively.

## Discussion

The main findings of this AIDA sub study are:

1. We found no difference in Lesion-oriented Composite Endpoint between oversized and non-oversized Absorb BVS treated lesions.
2. Definite Scaffold Thrombosis and Target Vessel Myocardial Infarction occurred more frequently in non-oversized Absorb BVS treated lesions, especially between 1 and 2 year follow-up.
3. Most cases of definite scaffold thrombosis occurred in properly sized Absorb BVS according to the Instructions for Use of Absorb BVS and Dmax assessment.
4. We found no difference in lesion-oriented outcomes between oversized and non-oversized Xience EES treated lesions.

Scaffold thrombosis is an unpredictable severe complication, which usually results in myocardial infarction, or even cardiac death. Several studies have investigated whether Absorb BVS optimized implantation techniques are able to resolve the scaffold thrombosis issue. The results of these studies are, however, equivocal. Correct scaffold sizing remains a challenge in routine PCI because appropriate device selection relies on the device to coronary diameter ratio and its potential mismatch [12, 17]. Correct scaffold sizing is particularly difficult in long tapered lesions. It is known that the avoidance of a scaffold to coronary diameter mismatch is associated with less frequent ischemia driven TLR, whereas scaffold oversizing may be associated with higher rates of MACE at 1 year follow-up [12]. Whether this theory also applies to metallic stents remains unknown.

In the current analysis we investigated the relationship between Dmax based device sizing and lesion-oriented outcomes in both the Absorb BVS as the Xience EES treated arm of the AIDA trial. Dmax based device sizing is a lesion specific characteristic, and therefore we present specific lesion-oriented outcomes. In contrast with the findings of Ishibashi et al, our analysis showed that oversized Absorb BVS were not associated with higher rates of lesion oriented events. This may be due to the difference in MI definition in the two studies, namely in AIDA trial there was no routine post-procedure marker measurement and only clinically significant post-procedural myocardial infarctions were captured. However, from 1 to 2 year follow-up, similarly to Katagiri et al. we found significant increase in the rates of device thrombosis in non-oversized Absorb BVS treated lesions. Furthermore, most cases of very late scaffold thrombosis occurred in properly sized devices [13]. This later finding may implicate the complex interaction between vessel wall and the device resorption and dismantling process rather that any initial procedural technique as a major factor in very late stent thrombosis cases.

Our study is the first to investigate the effect of Dmax based device sizing in Xience EES treated lesions. We found no difference in event rates between oversized and non-oversized Xience EES treated lesions. Definite stent thrombosis is unpredictable and does not depend on the Xience EES / coronary artery diameter mismatch. Moreover, all three acute stent thrombosis occurred in properly sized devices, in patients taking dual antiplatelet therapy, suggesting that other factors than implantation technique play a role in the occurrence. These results can potentially be attributed to the better device characteristics of the Xience EES such as thinner struts, better expansion limits, higher tensile and radial strength as compared to Absorb BVS.

The insights of this AIDA sub-study can be useful for the development of the next generation coronary bioresorbable scaffolds, with wider expansion limits and better tensile and radial strength, potentially resolving the scaffold sizing issues and difficulties. In addition, QCA or intravascular imaging should be strongly considered in forthcoming trials of new generation bioresorbable coronary scaffolds.

## Limitations

The current analysis has several limitations. First, in AIDA, device sizing based on online QCA was not mandatory, and therefore incorrect device sizing is likely to have occurred more frequently. Second, routine intravascular imaging has not been performed and therefore the study does not provide mechanistic insights in the occurrence of events due to a device / artery diameter mismatch. Third, the role of lesion morphology and typology stratification as a prognostic facture for device failure has not been analyzed in the current explorative analysis. The gold standard for lesion morphology and typology stratification is intracoronary imaging. The AIDA population reflects routine PCI, and since intracoronary imaging is not a part of routine PCI (used in less than 10% of the cases), it was not possible to explore the exact role of these factors in this study. Fourth, as with all post-hoc analysis, this AIDA sub-study is subject to under powering.

## Conclusions

In this AIDA trial sub-study, we found no significant difference in LOCE between oversized and non-oversized treated Absorb BVS and Xience EES treated lesions. However, non-oversized Absorb BVS implantation was associated with a higher risk of scaffold thrombosis at complete 2 years follow-up, in particular between 1 and 2 years follow-up.

## Electronic supplementary material

Below is the link to the electronic supplementary material.

Supplementary material 1 (DOCX 198 KB)

## Abbreviations

Absorb BVS: Absorb bioresorbable vascular scaffold
Dmax: Maximum diameter
LOCE: Lesion oriented composite endpoint
MACE: Major adverse cardiac events
MLD: Minimum lumen diameter
PCI: Percutaneous coronary revascularization
TLF: Target lesion failure
TLR: Target lesion revascularization
TVF: Target vessel failure
TV-MI: Target vessel myocardial infarction
QCA: Quantitative coronary angiography
Xience EES: Xience everolimus eluting stent
