# Supplementary material for: The relationship of pre-procedural Dmax based sizing to lesion level outcomes in Absorb BVS and Xience EES treated patients in the AIDA trial
Source: Int J Cardiovasc Imaging. 2019 Mar 25;35(7):1189–98. doi: 10.1007/s10554-019-01576-y (PMC6598967; doi:10.1007/s10554-019-01576-y)
Supplement: Supplementary file 1 — Supplementary material 1 (DOCX 198 KB) [file 10554_2019_1576_MOESM1_ESM.docx]

**Supplementary table legends.**

**Supplementary table 1:** Shown are the predictors of LOCE at 2 years after Absorb BVS implantation. LOCE = lesion oriented composite endpoint; BVS = bioresorbable vascular scaffold; HR = Hazard Ratio; MLD = minimum lumen diameter; RVD = reference vessel diameter; CI = confidence interval.

**Supplementary table 2.** Shown are the predictors of LOCE at 2 years after Xience EES implantation. LOCE = lesion oriented composite endpoint; EES = everolimus eluting stent; HR = Hazard Ratio; MLD = minimum lumen diameter; RVD = reference vessel diameter; CI = confidence interval .

**Supplementary table 1**

| Absorb BVS treated lesions | Univariate Cox regression | | | Multivariate Cox regression | | |
| --- | --- | --- | --- | --- | --- | --- |
|  | HR | (95% CI) | P-value | HR | (95% CI) | P-value |
| Device Oversize | 0.91 | (0.58 – 1.42) | 0.682 | 0.91 | (0.56 – 1.47) | 0.687 |
| Age – years | 1.03 | (1.01 – 1.05) | 0.008 | 1.02 | (1.00 – 1.05) | 0.074 |
| Male sex | 2.23 | (1.21- 4.11) | 0.011 | 2.48 | (1.28 – 4.79) | 0.007 |
| Current Smoker | 1.34 | (1.00 – 1.80) | 0.053 | 1.16 | (0.83 – 1.62) | 0.382 |
| Hypertension | 0.68 | (0.43 – 1.06) | 0.088 | 0.87 | (0.53 – 1.41) | 0.561 |
| Hypercholesterolemia | 1.00 | (0.63 – 1.58) | 0.995 |  |  |  |
| Any diabetes mellitus | 0.71 | (0.42 – 1.20) | 0.197 |  |  |  |
| Chronic Total Occlusion | 1.66 | (0.41 – 6.77) | 0.477 |  |  |  |
| Bifurcation Lesion | 1.37 | (0.43 – 4.35) | 0.591 |  |  |  |
| Severe or moderate calcification | 1.60 | (1.02 – 2.53) | 0.042 | 1.41 | (0.87 – 2.30) | 0.166 |
| Ostial Lesion | 0.56 | (0.26 – 1.22) | 0.143 |  |  |  |
| Thrombus present | 1.65 | (0.76 – 3.59) | 0.205 |  |  |  |
| TIMI 0 or 1 flow pre-PCI | 0.60 | (0.28 – 1.31) | 0.203 |  |  |  |
| Syntax Score | 1.04 | (1.02 – 1.06) | <0.001 | 1.03 | (1.01 – 1.05) | 0.005 |
| Pre-procedural diameter stenosis | 0.99 | (0.98 – 1.00) | 0.080 | 0.99 | (0.98 – 1.00) | 0.109 |
| Pre-dilatation performed | 1.75 | (0.55 – 5.55) | 0.340 |  |  |  |
| Pre-procedural in segment MLD | 1.00 | (0.63 – 1.59) | 0.991 |  |  |  |
| Pre-procedural in segment RVD | 0.85 | (0.58 – 1.24) | 0.392 |  |  |  |
| Amount stents implanted | 1.00 | (0.61 – 1.64) | 1.000 |  |  |  |
| Post-dilatation performed | 0.82 | (0.47 – 1.42) | 0.475 |  |  |  |
| Post-procedural percentage diameter stenosis | 1.01 | (0.99 – 1.03) | 0.484 |  |  |  |
| Post-procedural in segment MLD | 0.66 | (0.42 – 1.05) | 0.077 | 0.67 | (0.40 – 1.09) | 0.107 |

# Supplementary table 2

| Xience EES treated lesions | Univariate Cox regression | | | Multivariate Cox regression | | |
| --- | --- | --- | --- | --- | --- | --- |
|  | HR | (95% CI) | P-value | HR | (95% CI) | P-value |
| Device Oversize | 1.11 | (0.65 – 1.89) | 0.703 | 1.03 | (0.60 – 1.76) | 0.915 |
| Age – years | 1.00 | (0.97 – 1.02) | 0.689 |  |  |  |
| Male sex | 0.81 | (0.46 – 1.45) | 0.478 |  |  |  |
| Current Smoker | 1.15 | (0.82 – 1.62) | 0.423 |  |  |  |
| Hypertension | 0.63 | (0.37 – 1.08) | 0.094 | 0.58 | (0.33 – 1.01) | 0.054 |
| Hypercholesterolemia | 1.80 | (1.00 – 3.25) | 0.050 | 2.28 | (1.24 – 4.21) | 0.008 |
| Any diabetes mellitus | 0.53 | (0.30 – 0.95) | 0.032 | 0.553 | (0.30 – 1.01) | 0.053 |
| Chronic Total Occlusion | 0.80 | (0.20 – 3.28) | 0.755 |  |  |  |
| Bifurcation Lesion | 1.67 | (0.41 – 6.86) | 0.475 |  |  |  |
| Severe or moderate calcification | 1.73 | (1.01 – 2.95) | 0.044 | 1.58 | (0.92 – 2.71) | 0.097 |
| Ostial Lesion | 0.79 | (0.29 – 2.19) | 0.652 |  |  |  |
| Thrombus present | 2.04 | (0.74 – 5.63) | 0.170 |  |  |  |
| TIMI 0 or 1 flow pre-PCI | 0.76 | (0.32 – 1.76) | 0.516 |  |  |  |
| Syntax Score | 1.03 | (0.99 – 1.06) | 0.111 |  |  |  |
| Pre-procedural diameter stenosis | 0.99 | (0.98 – 1.01) | 0.289 |  |  |  |
| Pre-dilatation performed | 0.66 | (0.21 – 2.12) | 0.486 |  |  |  |
| Pre-procedural in segment MLD | 0.70 | (0.36 – 1.36) | 0.292 |  |  |  |
| Pre-procedural in segment RVD | 0.72 | (0.47 – 1.10) | 0.124 |  |  |  |
| Amount stents implanted | 1.53 | (0.89 – 2.65) | 0.127 |  |  |  |
| Post-dilatation performed | 0.64 | (0.38 – 1.09) | 0.104 |  |  |  |
| Post-procedural percentage diameter stenosis | 0.99 | (0.97 – 1.01) | 0.304 |  |  |  |
| Post-procedural in segment MLD | 0.86 | (0.50 – 1.45) | 0.565 |  |  |  |

**Supplementary figure legends.**

**Supplementary figure 1:** Shown are the ROC curves of the relative difference between the Dmax and the device diameter and LOCE at 2 years after Absorb BVS implantation (A) and Xience EES implantation (B). LOCE = lesion oriented composite endpoint; BVS = bioresorbable vascular scaffold; EES = everolimus eluting stent.

**Supplementary figure 2:** Shown are the ROC curves of the relative difference between the Dmax and the device diameter and definite device thrombosis at 2 years after Absorb BVS implantation (A) and Xience EES implantation (B). LOCE = lesion oriented composite endpoint; BVS = bioresorbable vascular scaffold; EES = everolimus eluting stent.


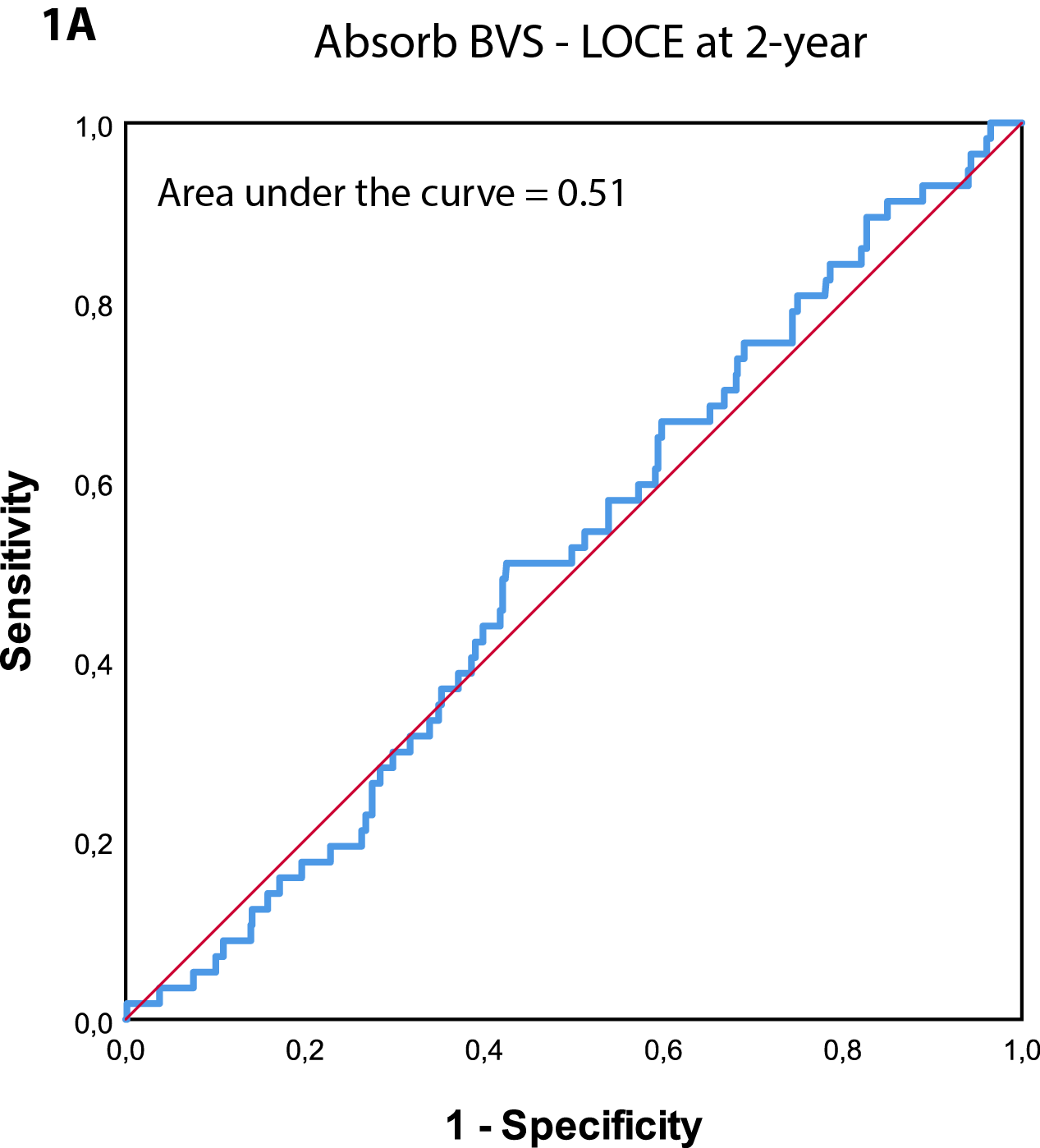

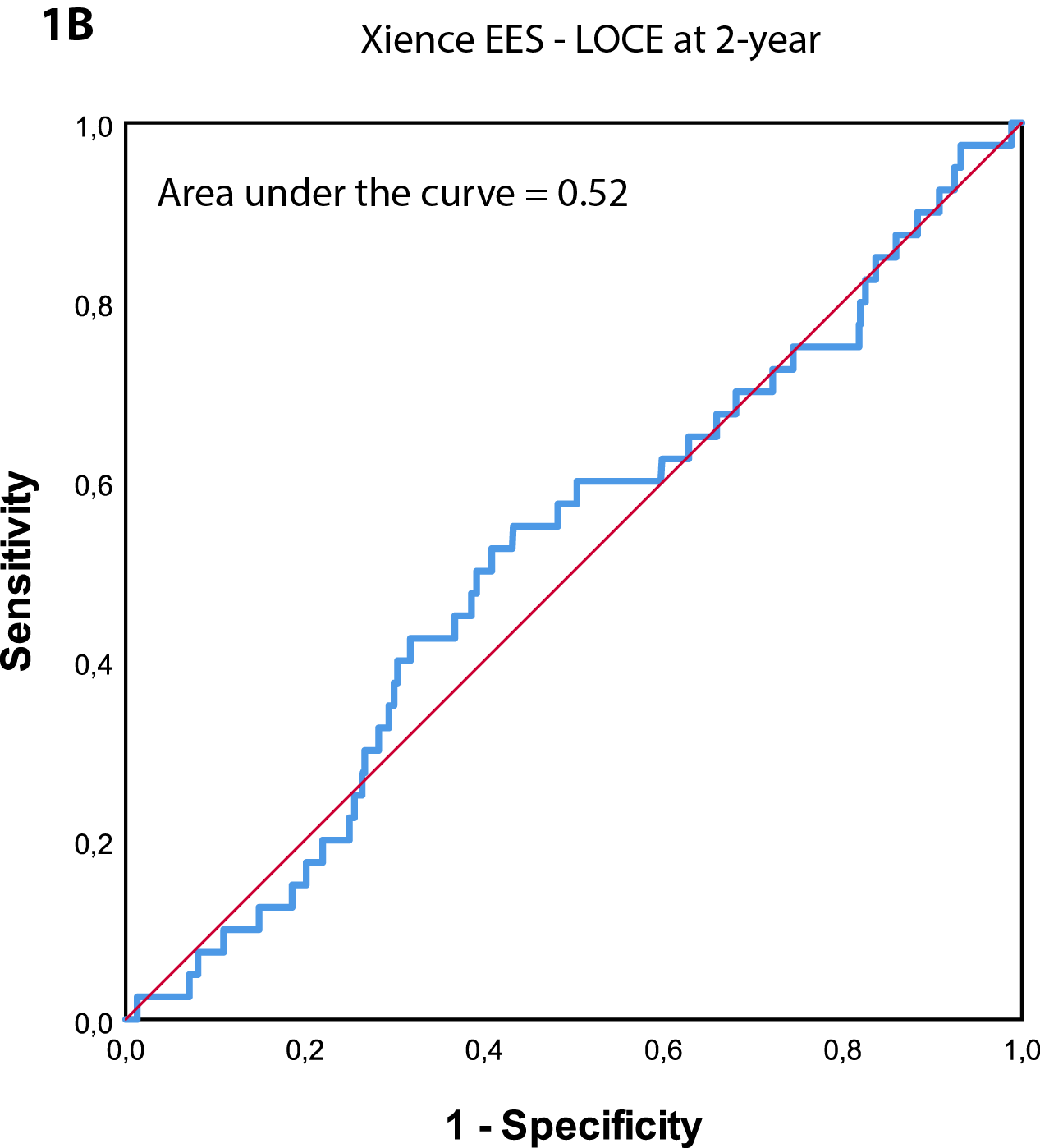


**
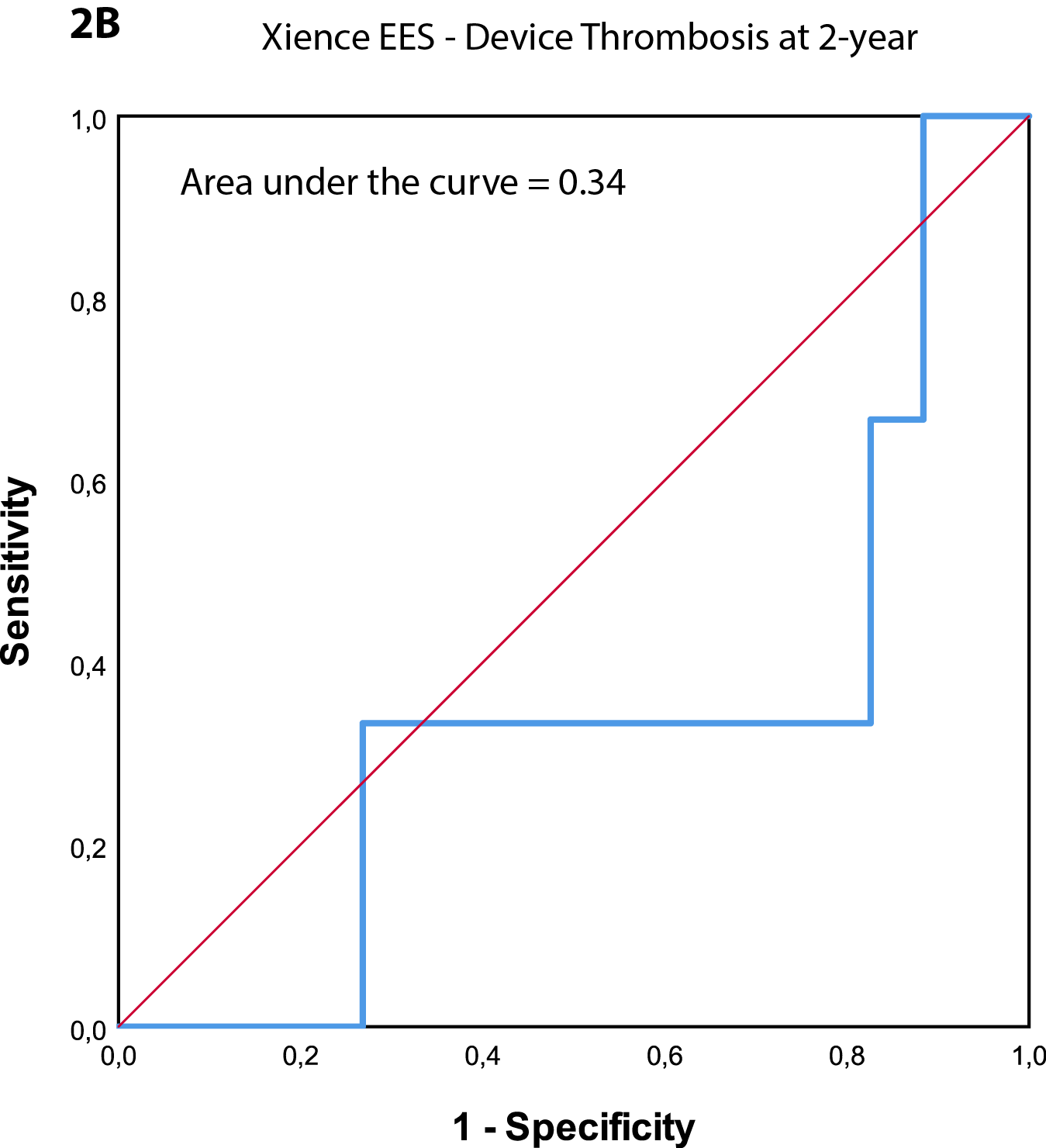

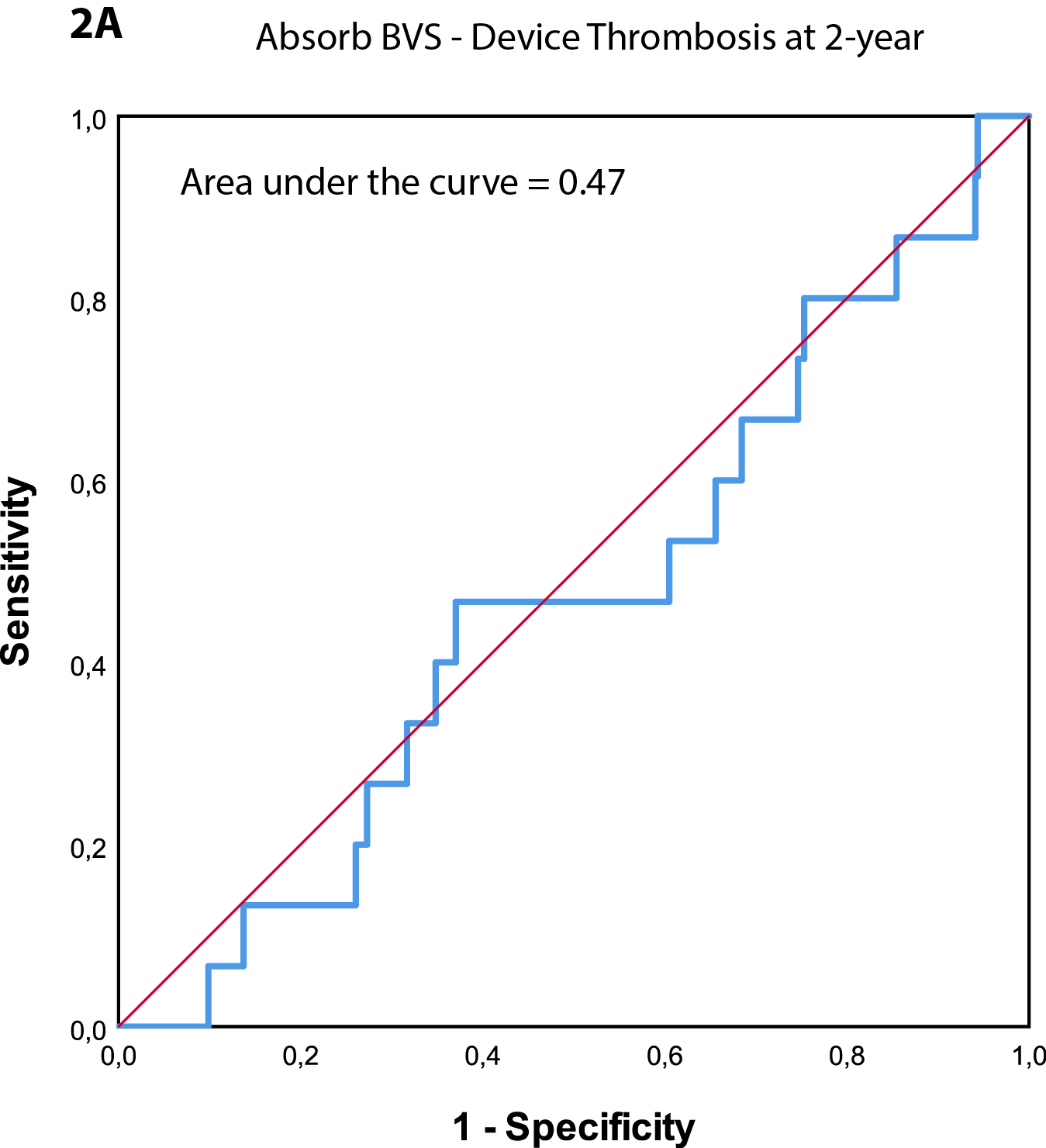
**
